# Chicago Medical College Clinic

**Published:** 1877-08

**Authors:** S. O. Richey


### CHICAGO MEDICAL COLLEGE CLINIC.

### Service of Prof. S. J. Jones.

[Reported by S. O. Richey, M. D.]

*A Case of Clonic Blepharo-Spasmus.*

February 28th, 1877. Mr. C., a railroad employe, has been a clerk for fifteen years past, working continually, much of the time by artificial light. Hard work, inability to get sufficient sleep to recuperate his strength, and an immoderate use of tobacco combined to produce marked nervous depression.

In both eyes vision is normal ($v=\frac{20}{20}$).

There is violent spasmodic action of the upper lids of both eyes, which, besides the annoyance it gives, interferes with the performance of his duties, because his eyelids will close at the most critical time.

He is advised to secure all the sleep possible, to eat good nutritious food, to rest the eyes, and to give up the use of tobacco and stimulating drinks. A shower bath for the eyes, and the elixir of calisaya, iron and strychnia are prescribed for him, with twenty grains (gr. xx) of bromide of potash, daily, when going to bed.

March 2d. He returns improved; the spasm of the lids has ceased, he sleeps better, eats more heartily and seems more vigorous.

March 5th. The improvement continues, the change being perceptible even in his complexion.

March 20th. The patient has been seen several times since the 5th inst., each time being stronger than before. He has had no return of the spasm.

The spasm of *both* lids, the absence of local inflammation, with the other symptoms mentioned, directed attention to the condition of the general system as the cause of the affection, the spasm being a local evidence of general nervous debility.

